# Neoadjuvant chemoradiation versus perioperative chemotherapy followed by surgery in resectable adenocarcinomas of the esophagogastric junction: A retrospective single center analysis

**DOI:** 10.3892/ol.2013.1709

**Published:** 2013-11-27

**Authors:** BJÖRN SCHULZE, DOMINIK BERGIS, PANAGIOTIS BALERMPAS, JÖRG TROJAN, GUIDO WOESTE, WOLF OTTO BECHSTEIN, CLAUS RÖDEL, CHRISTIAN WEISS

**Affiliations:** 1 Department of Radiation Oncology, Johann Wolfgang Goethe University Hospital, Frankfurt 60590, Germany; 2 Department of Gastroenterology, Johann Wolfgang Goethe University Hospital, Frankfurt 60590, Germany; 3 Department of General, Visceral and Transplantation Surgery, Johann Wolfgang Goethe University Hospital, Frankfurt 60590, Germany

**Keywords:** adenocarcinoma, esophagogastric junction, surgery, neoadjuvant chemoradiation, perioperative chemotherapy, multimodality treatment

## Abstract

The current study presents a retrospective comparison, performed at a single academic center, of preoperative chemoradiation (CRT) and perioperative chemotherapy (CT) in addition to surgery in locally advanced but resectable adenocarcinoma of the esophagogastric junction (AEG). A total of 29 consecutive patients with locally advanced AEGs were retrospectively analyzed. Treatment consisted of preoperative CRT (mean dose, 45.0 Gy) plus two cycles of CT with cisplatin and 5-FU or perioperative CT with epirubicin, cisplatin and capecitabine (three cycles preoperatively and postoperatively). Within four to six weeks following preoperative treatment, surgical therapy was performed. Median overall survival was 21.0 months in the perioperative CT group versus 41.7 months in the CRT group [P=0.36; hazard ratio (HR), 1.50; 95% confidence interval (CI), 0.58–3.84]. Three-year survival rates were 55 and 38%, respectively, in favor of the CRT group, and progression-free survival was 20.0 months in the CT group compared with 24.1 months in the CRT group (P=0.71; HR, 1.19; 95% CI, 0.46–3.05). The total number of major surgical complications was almost equal in the two groups. Margin-free resections were achieved in all patients of the CRT group, but only 76.9% of the CT group (P=0.05). In addition, significantly higher R0 resection rates and an increased number of pathological complete remissions were demonstrated in the CRT group compared with those of the CT group. These results appear to indicate a trend for improved progression-free and overall survival for the CRT group. As postoperative morbidity and mortality rates were similar in the two groups, the results support the use of CRT for patients with advanced AEG tumors.

## Introduction

The incidence of adenocarcinomas of the distal esophagus and esophagogastric junction (AEG) is rising notably in Western populations with >480,000 new cases diagnosed annually, accounting for 400,000 mortalities per year (1,2). Despite adequate preoperative staging and improvements in perioperative treatment, the overall prognosis remains poor, with a five-year-survival rate of ~40%. In addition, <30% of patients exhibit potentially operable tumors and the majority of patients already have locally advanced tumor stages with involvement of locoregional lymph nodes on presentation (3). For patients undergoing surgery following neoadjuvant therapy [chemoradiotherapy (CRT) or chemotherapy (CT) alone], three-year survival rates vary between 22 and 55% (4–11). To improve long-term survival rates, multimodal treatment strategies, including preoperative CT and neoadjuvant CRT, for locally advanced AEGs have been considered and investigated widely. In an updated meta-analysis, Sjoquist *et al* summarized the results of multimodal treatment strategies indicating a trend in favor of neoadjuvant CRT (12). In addition, a current phase III trial from the Netherlands has confirmed the feasibility and superiority of a neoadjuvant CR regimen compared with surgery alone (10). However, a standard of care for patients with AEG tumors has not yet been defined. Recently, the EORTC expert panel voted in favor of preoperative CRT for AEG I and II tumors and recommended perioperative CT for AEG III tumors (13).

The current study presents a retrospective analysis of a single center experience with preoperative CRT or perioperative CT in addition to surgery in locally advanced but resectable AEG. The aim of the study was to identify the advantages and potential disadvantages of the two treatment regimens. Patients who were treated either with perioperative CT or neoadjuvant CRT between the years 2006 and 2012 at the Johann Wolfgang Goethe University Hospital (Frankfurt, Germany) were included in the analysis. Major surgical and non-surgical complications were evaluated and a Kaplan-Meier survival analysis was performed to compare the overall and progression-free survival-estimates between the two groups.

## Patients and methods

### Patients and treatment

A retrospective analysis was performed of patients with advanced but resectable AEG, treated in neoadjuvant intention with neoadjuvant CRT or perioperative CT between January 2006 and October 2012. Patients were allocated to the two treatment regimens almost equally by the consensus decision of a multidisciplinary tumor board of the Johann Wolfgang Goethe University Hospital. In total, 29 patients were identified who received identical CRT or CT schedules. Patients with different treatment regimens or who were participants in clinical trials were excluded from the analysis to achieve a homogeneous study population. The study was approved by the ethics committee of the Johann Wolfgang Goethe University Hospital. Written informed consent was obtained from the patients.

### Preoperative staging

Initial staging included endoscopy of the upper gastrointestinal tract with multiple biopsies, computed tomography scan of the thorax and abdomen and an endoscopic ultrasound. Other diagnostics, including positron emission tomography/computed tomography or diagnostic laparotomy, were optional. Physical examination and laboratory testing were routinely performed in all patients.

### CRT group

The CRT group included 16 patients. Radiation therapy planning was based on three-dimensional computed tomography scans of the chest and upper abdomen with a resolution of 3-mm slice reconstructions. The planning target volume was delineated as the macroscopic gross tumor volume plus the safety margins of 15 mm in the circumferential, 30 mm in the oral and 50 mm in the aboral extension. Patients received a median cumulative dose of 45.0 Gy (range, 45.0–50.4 Gy) in single fractions of 1.8 Gy/day. In addition, patients received two cycles of cisplatin and 5-Fluorouracil (5-FU) in the first and fifth week of radiotherapy. Cisplatin was administered at a dose of 20 mg/m^2^ from day one to five of each cycle. 5-FU was administered at a dose of 600 mg/m^2^ as a continuous infusion from day one to five of each cycle.

### CT group

The CT group included 13 patients who received a maximum of six three-week cycles of epirubicin, cisplatin and capecitabine (three cycles preoperatively and postoperatively). On day one of every three-week cycle, 50 mg/m^2^ epirubicin was administered to each patient, followed by the administration of 60 mg/m^2^ cisplatin. Between days one and 14, 1,000 mg/m^2^ capecitabine was orally administered twice daily. Following surgical resection, patients underwent adjuvant treatment with the same CT regimen.

### Surgery and follow-up

Surgery in the two groups was performed between four and six weeks following preoperative treatment. Extended gastrectomy and distal esophagectomy, with Roux-en-Y esophagojejunostomy and two-field D2-lymph node dissection, was performed in patients with AEG II/III. Transthoracic esophagectomy and proximal gastrectomy with *en bloc* removal of the esophagus and adjacent lymph nodes were performed in patients with AEG I. Patients were first seen for physical examination six to eight weeks following the termination of therapy. Technical examination (esophagogastroduodenoscopy or computed tomography) was performed at the discretion of the attending physician. For follow-up, patients were observed every three months in the first year and every six months in the following years. Medical reports and information from the attending physicians were also taken into account for analysis.

### Statistical analysis

Data were analyzed and compiled using BiAS software for Windows (version 9.11; Epsilon-Verlag, Darmstadt, Germany), SPSS version 20 (SPSS, Inc., Chicago, IL, USA) and GraphPad Prism 5 for Windows (version 5; GraphPad Software Inc., La Jolla, CA, USA). P<0.05 was considered to indicate a statistically significant difference. Follow-up time was defined as the time between initiation of preoperative therapy and mortality or final contact. The primary endpoint of the study was overall survival (OS), calculated between initiation of preoperative therapy and mortality. Progression-free survival (PFS) was calculated between the initiation of neoadjuvant treatment and reported initial reaction (defined as locoregional relapse or distant metastases) or mortality.

Acute hematological side effects were recorded according to Common Toxicity Criteria, version 3.0 (http://ctep.cancer.gov/protocolDevelopment/electronic_applications/ctc.htm). For TNM staging, the current TNM classification at diagnosis was used respecting the 2009 revision of TNM classification for esophageal cancer (www.uicc.org).

## Results

### Patient follow-up

Median follow-up was 25.5 months (range, 6.0–73.3 months) in the CRT group versus 22.0 months in the CT group (range, 5.3–64.7 months). Patients and tumor characteristics are shown in Table I.

### Acute side effects and feasibility of perioperative and preoperative therapy

In the two groups, no acute non-hematological adverse events ≥grade 3, leading to treatment modifications, were recorded. Acute hematological toxicity grade 3 or 4 was reported more frequently in the CRT group [eight of the 16 patients (50%)] compared with the CT group [two of the 13 patients (15%)] (P=0.02). Therefore, a dose reduction of CT was necessary in 50% of CRT patients and 15% of CT patients. In 15 of the 16 patients (94%) in the CRT group and 12 of the 13 patients (92%) in the CT group, all scheduled preoperative CT cycles were able to be administered. In the CT group, eight of the 13 patients (62%) were unable to receive adjuvant CT due to prolonged hematological toxicity or deterioration of general condition (Table II).

### Major surgical and non-surgical postoperative complications and associated mortality

Postoperative pulmonary and pleural complications, including pneumonia, pneumothorax, relevant pleural-effusion and pleural empyema, occurred more frequently in the CRT group than in the CT group (44 vs. 8%, respectively; P=0.04). One patient in each group succumbed to their condition during hospitalization due to septic complications following anastomotic leakage (Table III). The two groups did not differ significantly in the number of major surgical complications (CRT group, 69% vs. CT group, 77%; P=0.63; Table IV).

### Pathological complete remission and rate of R0 resection

Pathological complete remission (pCR) of the tumors was observed in three patients in the CT group (CT group, 19% vs. CRT group, 0%; P=0.11). In addition, the R0 resection rate (complete tumor-free resection margins) was significantly higher in the CRT group compared with the CT group (100 vs. 77%, respectively; P=0.05; Table IV).

### OS and PFS

**Figure 1:**
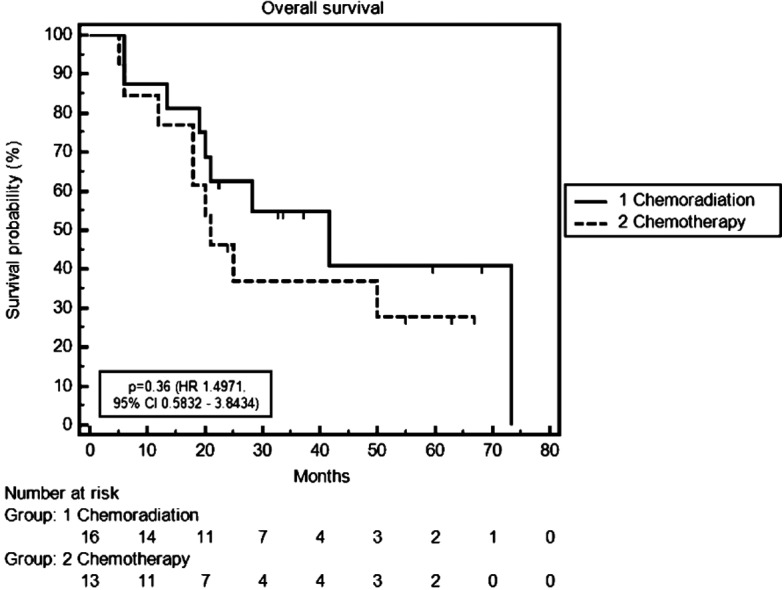
Overall survival probability for the two groups (neoadjuvant chemoradiation and perioperative chemotherapy) figured as Kaplan-Meier survival curves. There was no significant difference among the two groups (log rank test, P=0.36). HR, hazard ratio; CI, confidence interval.

**Figure 2:**
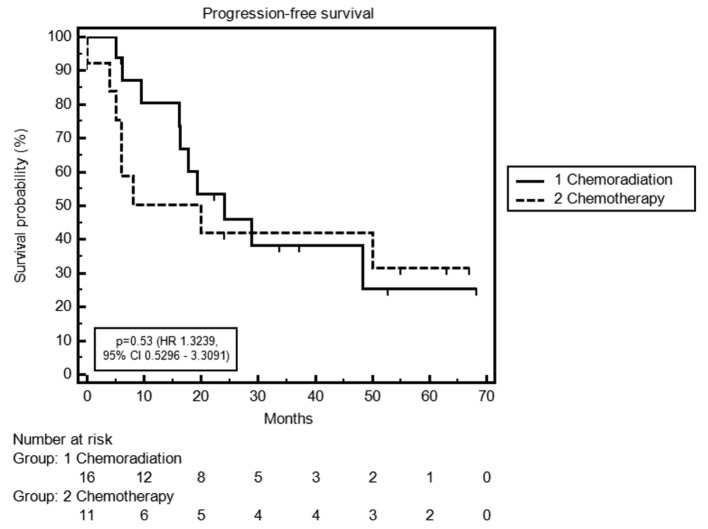
Progression-free survival probability for the two groups (neoadjuvant chemoradiation and perioperative chemotherapy) figured as Kaplan-Meier survival curves. There was no significant difference among the two groups (log rank test, P=0.71). HR, hazard ratio; CI, confidence interval.

Median OS time was 21.0 months in the CT group versus 41.7 months in the CRT group [P=0.36; hazard ratio (HR), 1.50; 95% confidence interval (CI), 0.58–3.84]. Three-year survival rates were 38% in the CT group and 55% in the CRT group. Median PFS time was 20.0 months in the CT group compared with 24.1 months in the CRT group (P=0.71; HR, 1.19; 95% CI, 0.46–3.05) (Table IV; Figs. 1 and 2).

### Patterns of recurrence and secondary malignancies detected during the follow-up period

The rate of local and/or distant recurrence during the follow-up period was 56% in the CRT and 69% in the CT group (Table IV). In the CRT group, three patients were diagnosed with secondary malignancies, including non-small cell lung cancer, colon carcinoma and cholangiocarcinoma. At least one patient in the CRT group succumbed to a secondary malignancy and not to the AEG tumor. In the other two cases, the cause of mortality was not clearly identifiable.

## Discussion

Although modern perioperative treatment regimens have shown improved outcomes for patients with advanced AEG tumors, there remains a lack of profound data to define the most effective treatment approach. The current study compared the outcome of a homogeneous patient population treated with preoperative CRT or perioperative CT at a single center. An updated meta-analysis in 2011 analyzing the administration of neoadjuvant CT or CRT in addition to surgery was unable to determine a clear advantage between the treatment regimens, although, a proportionately larger survival benefit was observed for CRT versus CT (12). However, concern remains that this benefit is achieved at the expense of an increase in morbidity and mortality. The present study found no significant differences in overall morbidity and mortality between the two treatment arms. This is consistent with the results of a number of randomized trials that found similar overall morbidity and mortality rates for perioperative CT and preoperative CRT when compared with surgery alone (Table V) (4–11,15–18). However, in the current study, a significantly higher rate of pulmonary complications (44%) was observed in the CRT compared with the CT group. Similar rates ranging between 20 and 50% have been reported by a number of authors investigating neoadjuvant CRT followed by surgery versus surgery alone. Notably, in these studies, the two groups (neoadjuvant CRT plus surgery vs. surgery alone) showed similar pulmonary morbidity rates. This indicates that pulmonary complications are not likely to be predominantly caused by the addition of radiotherapy or CT alone (6,8–10,19). However, the differences demonstrated in the current study are not well explained and highlight issues of target volume definition, radiotherapy dose/fractionation and lung sparing techniques, which must be accounted for in future studies. An additional difference between the treatment groups was observed in the frequency of hematological side effects, with 50% of the patients in the CRT group developing grade 3/4 hematotoxicity compared with only 15% in the CT group. The extremely low hematotoxicity in the CT group is contradictory to an additional single center phase II study investigating a similar preoperative CT regimen (epirubicin/cisplatin/capecitabine) that only differed by the dose of capecitabine. In this trial, the reported grade 3/4 neutropenia was 62% (20). By contrast, a recent phase III trial comparing preoperative CRT in addition to surgery with surgery alone reported considerably low rates of grade 3 and 4 hematotoxicity (<10%) with a treatment compliance of >90%, using a new chemotherapeutic regimen consisting of carboplatin and paclitaxel, with a radiation dose of 41.1 Gy in 1.8 Gy fractions. No differences in morbidity or mortality were observed in the two groups, however, patients in the CRT group showed significantly improved survival outcomes (10). Previously, the long-term results of the MRC OEO2 trial, which compared the additional effect of preoperative CT with surgery alone, showed that patients with microscopically complete resection (R0 resection) exhibited an OS rate of 42.4% compared with 18% of patients with microscopically incomplete resection (R1 resection) and 8.6% of patients with a remaining macroscopic tumor (R2 resection) (4). The present series identified a significantly higher R0 resection rate for patients who received CRT (100%) compared with those who received CT (77%). Furthermore, only patients in the CRT group (n=3) achieved pCR. Consecutively, a non-significant trend to a higher PFS and OS and an improved local control rate was identified in the CRT group. By contrast, preoperative CT was noted to decrease the incidence of distant metastasis by 31 versus 44% in the CRT group. Overall, these results reflect the results of the only two randomized trials that have directly compared preoperative CT with preoperative CRT. However, the two trials closed prematurely due to poor accrual. Stahl *et al* reported three-year OS rates of 47.4% in the CRT group and 27.7% in the CT group, with an increased number of patients in the CRT group experiencing pathological downstaging and pCR compared with the CT group (15.6 vs. 2%, respectively). Notably, all patients with pCR survived (11). Similarly, Burmeister *et al* was unable to demonstrate a significant survival benefit with the addition of radiation therapy to preoperative CT. The authors reported a prolonged time to progression, a significantly higher pCR rate and a trend to an improved R0 resection rate in the CRT group. The reported OS rates at three years were 49 (CT) versus 52% (CRT) and are consistent with the current results (6).

To determine the best multimodal treatment regimen, further studies are required. At present, an ongoing study, the international phase III TOPGEAR trial (launched in 2012), is investigating whether preoperative CRT (two cycles of ECF followed by 45 Gy of radiation with concurrent 5-FU) or preoperative CT (three cycles of ECF) alone is more effective in patients with resectable gastric and esophagogastric cancer. Following surgery, the two groups were scheduled to receive three additional cycles of ECF (unpublished data).

Despite the major limitations of the present small and retrospective analysis, the results confirmed the results of recent randomized trials addressing the issue of whether preoperative CT or CRT is superior for the treatment of AEG tumors. The current study demonstrated significantly higher R0 resection rates and an increased number of pCR in the CRT group. These results appear to indicate a trend for improved PFS and OS for the CRT group. As postoperative morbidity and mortality rates were similar in the two groups, the results of the current study support the use of CRT for patients with advanced AEG. Nevertheless, large trials integrating the best available treatment schedules are required to define a standard treatment approach for this increasingly common tumor entity.

## Figures and Tables

**Table I:** Patient and tumor characteristics.

Characteristics	Chemoradiotherapy	Chemotherapy
Patients, n	16 (100.0)	13 (100.0)
Gender		
Male	13 (81.0)	12 (92.0)
Female	3 (19.0)	1 (8.0)
Age, years		
Median (range)	63.8 (36.4–77.6)	58.8 (29.1–79.1)
<50	2 (13.0)	4 (31.0)
51–60	4 (25.0)	3 (23.0)
61–70	5 (31.0)	2 (15.0)
>70	5 (31.0)	4 (31.0)
Tumor site^a^		
AEG I	11 (69.0)	8 (62.0)
AEG II	3 (19.0)	3 (23.0)
AEG III	2 (12.0)	2 (15.0)
Preoperative T stage		
2	2 (12.5)	4 (31.0)
3	12 (75.0)	9 (69.0)
4	2 (12.5)	0 (0.0)
Preoperative N stage		
0	3 (19.0)	4 (31.0)
+	13 (81.0)	9 (69.0)

a AEG allocation determined by the anatomical localization of the tumor site according to Siewert’s classification (14).

Values are presented as n, (%), unless specified otherwise. AEG, adenocarcinoma of the esophagogastric junction.

**Table II:** Acute toxicity, treatment characteristics and comorbidity.

Treatment	Chemoradiotherapy	Chemotherapy
Patients	16 (100)	13 (100)
Acute toxicity		
Non-hematological	0 (0)	0 (0)
Hematological (CTC grade 3/4)	8 (50)^a^	2 (15)^a^
Cumulative dose of irradiation, Gy (range)	45.0 (45.0–66.6)^b^	NA
Preoperative Chemotherapy		
All scheduled cycles of chemotherapy	15 (94)	12 (92)
Dose reduction of chemotherapy during preoperative treatment	8 (50)	2 (15)
Postoperative Chemotherapy		
Receiving postoperative chemotherapy	NA	5 (38)

a P=0.02 (Fisher’s exact test);

b 1.8 Gy/fraction.

Values are presented as n, (%), unless specified otherwise. CTC, Common Terminology Criteria for Adverse Events of the National Cancer Institute (version 3.0); NA, not applicable.

**Table III:** Surgical complications and mortality.

Complication	Chemoradiotherapy, n (%)	Chemotherapy, n (%)	P-value^a^
Patients	11 (69)	10 (77)	NS
Type of major surgical complication			
Anastomotic leakage	4 (25)	4 (31)	NS
Mediastinitis/sepsis	1 (6)	1 (8)	NS
Implantation of esophageal stent	2 (13)	2 (15)	NS
Pulmonary complications	7 (44)	1 (8)	0.04
Secondary surgery	3 (19)	1 (8)	NS
Necrosis of intrathoracic gastric tube/neoesophagus	1 (6)	0 (0)	NS
Lymphatic fistula	1 (6)	0 (0)	NS
Other	5 (31)	3 (23)	NS
Complication-associated mortality	1 (6)	1 (8)	NS

a Determined by Fisher’s exact test.

NS, not significant.

**Table IV:** Surgical outcome and survival data.

Outcome	Chemoradiotherapy	Chemotherapy	P-value^a^
R0 resection^b^	16 (100)	10 (77)	0.05
pCR^c^	3 (19)	0 (0)	0.23
Median OS, months	41.7	21.0	0.36
3-year OS rate, %	55.0	38.0	NS
Median PFS, months	24.1	20.0	0.71
Pattern of recurrence			
Locoregional	2 (13)	4 (31)	NS
Distant	7 (44)	4 (31)	NS
Locoregional and distant	1 (6)	1 (8)	NS

a Determined by Fisher’s exact test;

b all resection margins clear in postoperative specimen;

c Absence of any viable tumor cells in postoperative specimen.

Values are presented as n, (%), unless specified otherwise. pCR, pathological complete regression; OS, overall survival; PFS, progression-free survival; NS, not significant.

**Table V:** Studies on multimodal treatment strategies in AEG.

A, Studies on perioperative CT followed by OP versus OP alone.				

		n/OS rate, %		
			
Author (year) [ref]	Years of accrual	CT and OP	OP	Postoperative toxicity
Kelsen *et al*(1998) [7]	1990–1995	124/23 (3-year)	120/26 (3-year)	No difference in morbidity/mortality
Allum *et al*(2009) [4] and MRC OEO2 (2009) [17]	1992–1998	265/22.6 (3-year)	268/17.6 (3-year)	No difference in morbidity/mortality
Cunningham *et al*(2006) [14]	1994–2002	65/38 absolute	66/31 absolute	No difference in morbidity/mortality
Ychou *et al*(2011) [15]	1995–2003	109/38 (5-year)	110/24 (5-year)	No difference in morbidity/mortality

B, Studies on preoperative CRT followed by OP versus OP alone.				

		n/OS rate, %		
			
Author (year) [ref]	Years of accrual	CRT and OP	OP	Postoperative toxicity

Urba *et al*(2001) [8]	1989–1994	37/30 (3-year)	38/16 (3-year)	No difference in morbidity/mortality
Walsh *et al*(1996) [9]	1990–1995	58/32 (3-year)	55/6 (3-year)	No difference in morbidity/higher mortality for CRT
Burmeister *et al*(2005) [5]	1994–2000	78/28 (3-year)	83/30 (3-year)	No difference in morbidity/mortality
Tepper *et al*(2008) [16]	1997–2000	23/39 (5-year)	19/16 (5-year)	No difference in morbidity/mortality
van Hagen *et al*(2012) [10]	2004–2008	134/~55^a^ (3-year)	141/~45^a^ (3-year)	No difference in morbidity/mortality

C, Studies on preoperative CRT followed by surgery versus preoperative CT followed by OP.				

		n/OS rate, %		
			
Author (year) [ref]	Years of accrual	CRT and OP	OP	Postoperative toxicity

Stahl *et al*(2009) [11]	2000–2005	60/47.4 (3-year)	59/27.7 (3-year)	Postoperative mortality was not significantly increased for CRT group
Burmeister *et al*(2011) [6]	2000–2006	39/52 (3-year)	36/49 (3-year)	No difference in morbidity/mortality
		39/45 (5-year)	39/36 (5-year)	

a Extracted from Kaplan-Meier survival curves.

AEG, adenocarcinoma of the gastroesophageal junction; CT, chemotherapy; CRT, chemoradiation; OP, surgery.
